# PD-1 Primarily Targets TCR Signal in the Inhibition of Functional T Cell Activation

**DOI:** 10.3389/fimmu.2019.00630

**Published:** 2019-03-29

**Authors:** Reina Mizuno, Daisuke Sugiura, Kenji Shimizu, Takumi Maruhashi, Mizuki Watada, Il-mi Okazaki, Taku Okazaki

**Affiliations:** Division of Immune Regulation, Institute of Advanced Medical Sciences, Tokushima University, Tokushima, Japan

**Keywords:** T cell receptor, co-receptor, PD-1, CD28, ICOS, cytokine production, follicular helper T cell

## Abstract

Cancer-immunotherapy targeting programmed cell death 1 (PD-1) activates tumor-specific T cells and provides clinical benefits in various cancers. However, the molecular basis of PD-1 function is still enigmatic. Especially, it is unclear which signaling pathway PD-1 primarily targets. Besides, the capacity of PD-1 to inhibit the T cell receptor (TCR)-dependent activation of T cells in the presence of co-stimulation is also controversial. Here we used co-culture systems of T cells and antigen-presenting cells with targeted deletion and overexpression of co-receptors and ligands and examined the inhibitory potency of PD-1 against T cell activation upon TCR stimulation with CD28 and ICOS co-stimulation. As an unambiguous criterion of T cell activation, we used the acquisition of cytokine production capacity, which represents one of the most important functions of T cells. PD-1 inhibited functional T cell activation upon TCR stimulation in the absence as well as in the presence of CD28 co-stimulation, indicating that PD-1 can directly inhibit TCR signal. Notably, CD28 co-stimulation rather attenuated the efficiency of PD-1 in inhibiting TCR-dependent functional T cell activation. In addition, PD-1 inhibited TCR-dependent functional T cell activation with ICOS co-stimulation as efficiently as that with CD28 co-stimulation. Furthermore, we found that the maintenance of antigen-induced follicular helper T (T_FH_) cells that required ICOS co-stimulation was persistently restrained by PD-1 *in vivo*. These findings indicate that PD-1 primarily targets TCR signal in the inhibition of functional T cell activation. Thus, PD-1 functions as the rheostat of T cell activation rather than an inhibitor of a specific stimulatory co-receptor.

## Introduction

T cell activation initiated by antigen-dependent signals through T cell receptors (TCRs) is tightly controlled by antigen-independent signals through stimulatory and inhibitory co-receptors to optimize beneficial immune responses while suppressing deleterious immune responses (1–3). Programmed cell death 1 (PD-1), one of the inhibitory co-receptors plays especially important roles in the regulation of autoimmunity, infectious immunity, and cancer immunity. PD-1 deficient mice develop various forms of autoimmune diseases spontaneously depending on their genetic background and lethal inflammatory diseases upon infection with several pathogens that establish chronic infection in PD-1-sufficient mice (4–7). In addition, cancer-immunotherapy targeting PD-1 activates tumor-specific T cells and eradicate tumors in animal models as well as human patients with various cancers (8, 9). Although the augmentation of T cell activation by PD-1 blockade is widely accepted, its molecular basis remains elusive.

PD-1 has two ligands, PD-L1 and PD-L2 that are expressed on a variety of immune and non-immune cells. The engagement of PD-1 with either PD-L1 or PD-L2 during antigen stimulation leads to the phosphorylation of two tyrosine residues in the cytoplasmic region of PD-1, the recruitment of protein tyrosine phosphatase, SHP-2 to the distal phospho-tyrosine, and the decreased phosphorylation of various signaling molecules including CD3ζ, ZAP70, vav, Akt, and ERK in T cells and Igβ, Syk, PLCγ2, and ERK in B cells (10–13). Although PD-1 has been regarded to inhibit T cell activation by dephosphorylating these molecules by recruiting SHP-2, the recent report by Hui et al. demonstrated that PD-1-bound SHP-2 dephosphorylated CD28 but not CD3ζ and inducible T cell co-stimulator (ICOS), another stimulatory co-receptor using a cell-free assay system, making the molecular target(s) of PD-1-bound SHP-2 controversial (14).

There had been also controversial reports regarding the capacity of PD-1 to suppress T cell activation in the presence of co-stimulatory signals. Parry et al. reported that PD-1 inhibited CD28-mediated activation of phosphatidylinositol 3-kinase and the subsequent Akt phosphorylation (11). On the other hand, Bennet et al. reported that PD-1 could inhibit TCR-dependent T cell activation with ICOS but not CD28 co-stimulation (15). The reduced inhibitory function of PD-1 in the presence of CD28 co-stimulation was also observed in other studies (16, 17). In addition, most tumor cells, especially non-hematopoietic tumor cells do not express ligands of CD28, CD80, and CD86. Therefore, the inhibition of CD28 signal cannot be the mechanism how PD-1 suppresses cytotoxicity against tumor cells that express ligands of PD-1 but not those of CD28 (18). Thus, the inhibitory potency of PD-1 against T cell activation upon TCR stimulation with co-stimulation is currently ambiguous.

Although cell-free assay systems are useful for the precise analyses of the physical features of proteins, some important physiological properties of proteins including their subcellular localization, their association to other proteins, cytoskeletal components, and organelles, or their functional changes upon stimulation may not be preserved properly. In addition, the direct correlation of the transient changes in phosphorylation levels of proteins to T cell function is not fully demonstrated. T cell activation is a complex multistep process and the longer and/or serial encounter of antigens over the period of hours and days is required for the functional activation of T cells (19–23). The recent technical advancements including TALEN and CRISPR/Cas9 systems allow us to destroy or modify genes of interest with high efficiency (24–27). By using these systems, we can now directly explore the coordination of genes with high accuracy in live cells or animals using biological functions as readouts more easily than before.

Here we used co-culture systems of T cells and antigen-presenting cells (APCs) with targeted deletion and overexpression of co-receptors and ligands to evaluate their actual involvements in PD-1-dependent inhibition of functional T cell activation. As an unambiguous criterion of functional T cell activation, we used the acquisition of cytokine production capacity, which directly reflects T cell activation and represents one of the most important functions of T cells. Remarkably, PD-1 inhibited functional T cell activation upon TCR stimulation in the absence as well as in the presence of CD28 co-stimulation. Notably, CD28 co-stimulation rather attenuated the efficiency of PD-1 in inhibiting TCR-dependent functional T cell activation. In addition, PD-1 inhibited functional T cell activation with ICOS co-stimulation as efficiently as that with CD28 co-stimulation. Furthermore, we found that the maintenance of antigen-induced follicular helper T (T_FH_) cells that required ICOS co-stimulation was persistently restrained by PD-1 *in vivo*. These findings indicate that PD-1 primarily targets TCR signal in the inhibition of functional T cell activation.

## Materials and Methods

### Cell Culture

DO11.10 T hybridoma, 2B4.11 T hybridoma, IIA1.6, and CH27 cells were maintained in RPMI 1640 medium (Gibco), supplemented with 10% (v/v) fetal bovine serum (FBS, Biowest), 0.5 mM Monothioglycerol (Wako), 2 mM L-alanyl-L-glutamine dipeptide (Gibco), 100 U/ml penicillin (Nacalai Tesque), and 100 μg/ml streptomycin (Nacalai Tesque). Plat-E cells were maintained in Dulbecco's Modified Eagle Medium (D'MEM, Invitrogen), supplemented with 10% (v/v) FBS, 100 U/ml penicillin (Nacalai Tesque), and 100 μg/ml streptomycin (Nacalai Tesque).

### Plasmid and Retroviral Gene Transduction

Fragments of cDNA were amplified by PCR and cloned into retroviral expression plasmid vectors modified from pFB-ires-Neo (Agilent). For controlling the expression levels, fragments of cDNA were cloned into retroviral expression plasmid vectors modified from pSUPER.retro.puro (OligoEngine), the promoter region of which was replaced with EF-1α (human elongation factor-1 alpha) coupled with or without polyadenylation (pA) signal. Plasmids were transfected using the FuGENE HD (Promega) into Plat-E cells cultured in D'MEM high glucose (Gibco) supplemented with 20% (v/v) FBS, 100 U/ml penicillin (Nacalai Tesque), and 100 μg/ml streptomycin (Nacalai Tesque) and supernatants containing viruses were used to transduce genes into target cells. Infected cells were selected with G418 (Wako), puromycin (Sigma-Aldrich), or blasticidin (InvivoGen).

### Mice

BALB/c mice (Japan SLC) were housed under specific pathogen-free conditions in environmentally controlled clean rooms. All experimental procedures complied with institutional regulations complying with the Act on Welfare and Management of Animals and the related guidelines in Japan. All mouse protocols were approved by the Animal Experimentation Committee of Tokushima University.

### Flowcytometric Analysis

Cultured cells and primary T cells were stained with the indicated Abs. Data were obtained with Gallios (Beckman Coulter) and analyzed using FlowJo (Tree Star). Abs against Bcl6 (K112-91) and CXCR5 (2G8) were purchased from BD bioscience. Abs against CD3 (145-2C11), CD80 (16-10A-1), CD86 (GL-1), and PD-L2 (TY25) were purchased from eBioscience. The Ab against CD8 (5H10) was purchased from Thermo Fisher Scientific. Abs against CD3 (17A2), CD4 (RM4-5), CD28 (37.5), CD45 (30-F11), ICOS (C398.4A), ICOSL (HK5.3), PD-1 (RMP1-30), and PD-L1 (10F.9G2), Brilliant violet 421-conjugated streptavidin, armenian hamster IgG (HTK888), rat IgG2b (RTK4530), and syrian hamster IgG (SHG-1) were purchased from Biolegend. FoxP3 Transcription Factor Staining Buffer Kit (TONBO) was used for the staining of Bcl6.

### Stimulation of T Hybridoma Cells and Primary T Cells

DO11.10 and 2B4.11 T hybridoma cells were stimulated as described before (28). Briefly, indicated T hybridoma cells (5 × 10^4^ cells/well) were stimulated with indicated APCs (1 × 10^4^ cells/well) pulsed with indicated amount of cognate peptide (OVA_323−339_ (ISQAVHAAHAEINEAGR, >95% purity, Sigma-Aldrich Japan or eurofins Genomics) and MCC_88−103_ (ANERADLIAYLKQATK, >95% purity, TORAY) for DO11.10 and 2B4.11 T hybridoma cells, respectively) in 96-well round bottom plate (BD Biosciences) for 12 to 14 h. Where indicated, 0.5 μg/ml of anti-PD-L1 Ab (1-111A) (29) or mouse IgG2a isotype control (RTK2758, Biolegend) were added. Splenocytes and lymph node cells were stimulated with anti-CD3 Ab (0.3 μg/ml, 145-2C11) for 24 h. CD4^+^ and CD8^+^ T cells were purified by using biotinylated Abs against CD4 (RM4-5, Biolegend) and CD8α (53-6.7, Biolegend), respectively and Anti-Biotin MicroBeads (Miltenyi Biotec) (>90% purity). Pre-activated primary CD4^+^ T cells (2 × 10^4^ cells/well) were stimulated with indicated APCs (1 × 10^4^ cells/well) pulsed with indicated amount of anti-CD3 Ab (145-2C11) in 96-well round bottom plate for 36 h. Pre-activated primary CD8^+^ T cells (1 × 10^4^ cells/well) were stimulated with indicated APCs (5 × 10^3^ cells/well) pulsed with indicated amount of anti-CD3 Ab (145-2C11) in 96-well-round bottom plate for 36 to 48 h. The concentration of IL-2 and IFNγ in the culture supernatant was determined by ELISA (Biolegend and Thermo scientific, respectively).

### Targeted Gene Knockout

IIA1.6 cells deficient for CD86, ICOSL and PD-L1, CH27 cells deficient for CD80, CD86, and PD-L1, and DO11.10 T hybridoma cells deficient for CD28 were generated by using CRISPR/Cas9 system. Guide RNA sequences targeting individual genes (*Cd274*: 5′-GTATGGCAGCAACGTCACGA-3′; *Cd86*: 5′-GAGACGCAAGCTTATTTCAA-3′; *Cd80*: 5′- GTCCAAGTCAGTGAAAGATA-3′; *Icosl*: 5′-ACATGGAGCTTCTTCCAAAC-3′; *Cd28*: 5′-ACTCGGCATTCGAGCGAAAC-3′ and 5′-GCTGTTCACGCCCTTGTACA-3′) were cloned into pEF-BOS-Cas9-U6-guide, which was modified from pEF-BOS (30) to express humanized Cas9 cDNA (Addgene) under human EF-1α-promoter and guide RNA under U6 promoter in opposite directions. Plasmids were transfected into cells by electroporation (Nucleofector II, Lonza) and cells that have lost the expression of targeted genes were sorted by using cell sorter (MoFlo XDP, Beckman Coulter). Clones of cells were obtained by limiting dilution for IIA1.6-PD-L1KO, IIA1.6-PD-L1/CD86 double KO, CH27-CD80/CD86/PD-L1 triple KO, and DO11.10-CD28KO T hybridoma cells. IIA1.6-PD-L1/ICOSL double KO cells were obtained by targeting ICOSL gene in IIA1.6-PD-L1KO cells followed by cell sorting. The introduction of loss-of-function mutations in targeted genes and the lack their expression were confirmed by sequencing and flowcytometry, respectively.

### Quantitative PCR

Total RNA was extracted from indicated cells using TRIzol (Ambion), and then subjected to reverse transcription using High-Capacity cDNA Reverse Transcription Kit (Applied Biosystems). Gene expression was analyzed by quantitative PCR (qPCR) using Power SYBR Green PCR Master mix (Applied Biosystems) on a 7900HT Fast Real-time PCR (Applied Biosystems). Values were normalized to the expression of *Actb*. Nucleotide sequences of primer sets are listed in Table 1.

**Table 1:** Primer sets for quantitative PCR.

**Gene**	**Forward**	**Reverse**
*Klf2*	ACCAAGAGCTCGCACCTAAA	TCCTTCCCAGTTGCAATGAT
*Il4*	GGTCTCAACCCCCAGCTAGT	GCCGATGATCTCTCTCAAGTGAT
*Il21*	GCCAGATCGCCTCCTGATTA	CATGCTCACAGTGCCCCTTT
*Prkcq*	ACCCACCCTTCAGACCAAAA	GATAGCCGGGGTTTCTCACT
*Zap70*	CAAGCAGGGTAAGAGGATGGA	ACGTTGTTCCACAGTCAGGA
*Lat*	GAGCTGGCCTCTGTGAACTC	ACACAGACTGGACCCCAAAG
*Actb*	TCCAGCCTTCCTTCTTGGGTA	CAGCACTGTGTTGGCATAGAGG

### Analysis of T_FH_ Cells

BALB/c mice were immunized subcutaneously with NP-OVA (20 μg) emulsified in Immject alum (Thermo Fisher). On day 6 and 8 after immunization, mice were treated with indicated Ab (anti-ICOSL Abs (HK5.3, 300 μg, Bio X cell), anti-PD-L1 Abs (1-111A, 500 μg), or their combination) or control IgG (2A3, Bio X cell). CD19^−^CD3^+^CD4^+^ T cells from popliteal lymph nodes were analyzed by flowcytometery at day 9.

### Statistics

Two-tailed unpaired Student's *t*-test or one-way ANOVA with Tukey HSD test was used to evaluate statistical significance. *p* < 0.05 was considered statistically significant.

## Results

### Inhibition of IL-2 Production From DO11.10 T Cells by PD-1

**Figure 1:**
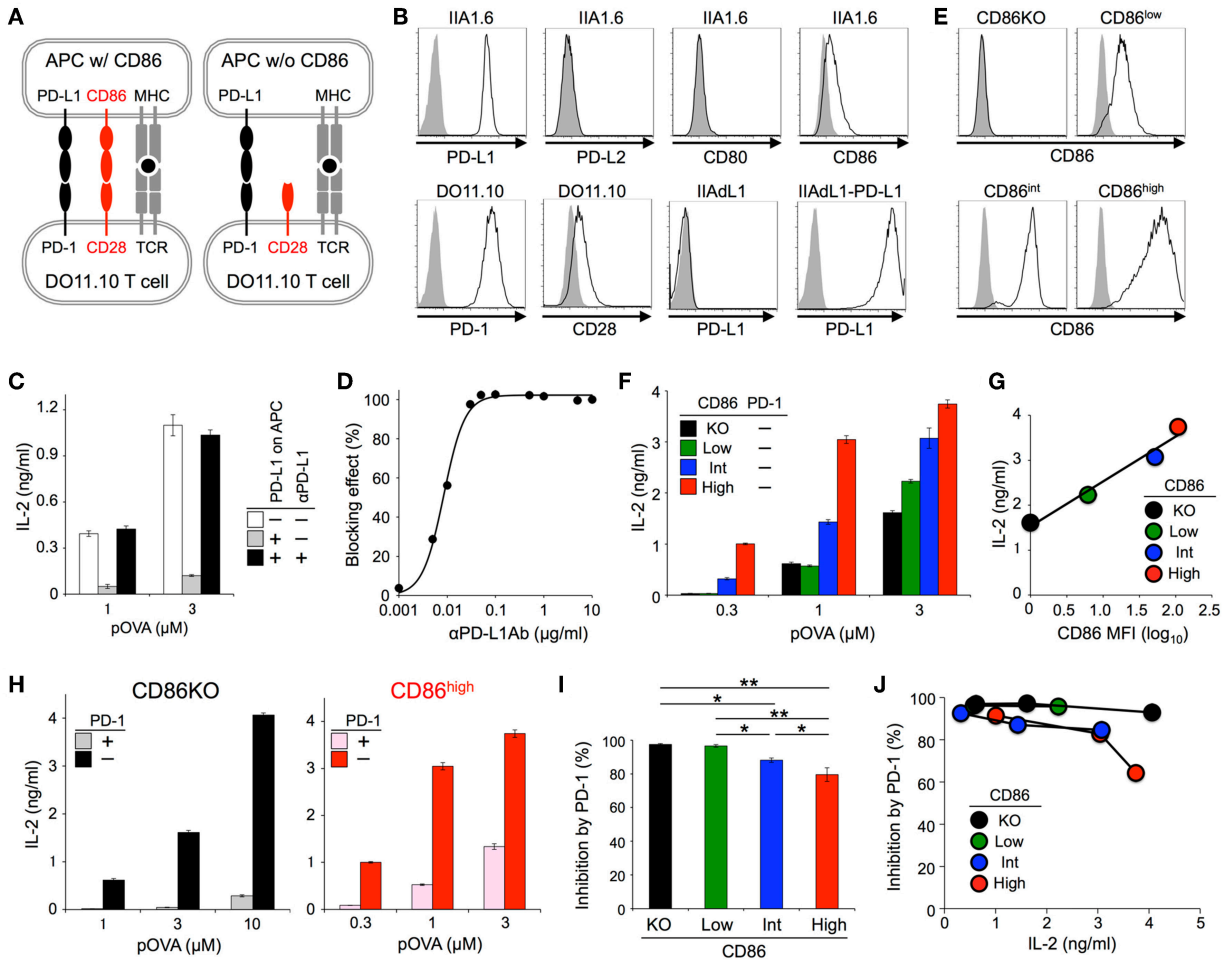
PD-1 inhibited the antigen-dependent functional activation of DO11.10 T cells less efficiently in the presence of CD28 co-stimulation. **(A)** Schematic representations of the antigen-dependent activation of DO11.10 T cells with or without CD28 engagement. **(B)** Expression levels of indicated co-receptors and ligands. **(C)** Inhibition of antigen-dependent activation of DO11.10 T cells by PD-1 engagement. IL-2 secretion from DO11.10 T cells in the absence (white) or presence (gray) of PD-1 engagement by PD-L1 on APCs. Anti-PD-L1 Ab completely blocked PD-1 effect (black). **(D)** Titration of anti-PD-L1 blocking Ab. **(E)** Expression levels of CD86 on IIA1.6 cells expressing CD86 to varying degrees. **(F)** Antigen-dependent activation of DO11.10 T cells in the absence of CD28 co-stimulation and the enhancement of the activation in a manner dependent on the amount of CD86 on APCs. **(G)** Correlation between the amount of secreted IL-2 and the expression level of CD86 on APCs. **(H–J)** Robust PD-1-mediated inhibition of IL-2 production from DO11.10 T cells in the absence CD28 co-stimulation and the partial attenuation of PD-1-mediated inhibitory effect by CD28 co-stimulation. IL-2 secretion from DO11.10 T cells upon stimulation with pOVA_323−339_-pulsed APCs lacking (left, black and gray) or expressing (right, red and pink) CD86 in the presence (gray and pink) or absence (black and red) of PD-1 engagement **(H)**. The average percent PD-1-dependent inhibition of IL-2 production upon stimulation with indicated APCs pulsed with 0.3, 1, and 3 μM of pOVA_323−339_ **(I)**. The percent PD-1-dependent inhibition is plotted in relation to the amount of IL-2 production in the absence of PD-1 engagement for indicated APCs **(J)**. Data are the mean ± SEM of technical triplicates in one experiment. Data are representative of more than two independent experiments. *p < 0.05 and **p < 0.01 by one-way ANOVA with Tukey HSD test. Cells used in this figure are listed in Tables 2, 3.

We first tried to test whether PD-1 exclusively targets CD28 signal or not in inhibiting functional T cell activation by using an *in vitro* experimental system that represents physiological antigen-dependent activation of T cells. We used DO11.10 hybridoma T cells that recognize 323-339 segment of chicken ovalbumin (pOVA_323−339_) in the context of I-A^d^ (Figure 1A; Tables 2, 3) (31, 32). Upon co-culturing with pOVA_323−339_-pulsed IIA1.6 B lymphoma cells that express I-A^d^, DO11.10 T cells were activated and secreted IL-2. Because the amount of secreted IL-2 correlated with the amount of pOVA_323−339_, we evaluated the strength of activation based on the amount of secreted IL-2. DO11.10 T cells endogenously expressed substantial amount of PD-1, whereas a low level of PD-L1 and no PD-L2 expression could be detected on IIA1.6 cells (Figure 1B). We knocked out PD-L1 gene in IIA1.6 cells by using CRISPR/Cas9 system to obtain IIA1.6-PD-L1KO (IIAdL1) cells. When we overexpressed PD-L1 in IIAdL1 cells and used them (IIAdL1-PD-L1 cells) as APCs for the stimulation of DO11.10 T cells, strong PD-1-mediated suppression of IL-2 production was observed (Figure 1C). Because this inhibitory effect was completely blocked by the addition of anti-PD-L1 Ab, we evaluated the inhibitory effect of PD-1 by comparing the presence or absence of anti-PD-L1 Ab, hereafter (Figures 1C,D).

**Table 2:** APCs used in this study.

	**PD-L1**		**PD-L2**		**CD80**		**CD86**		**ICOSL**		**FcγRIIB**		
	**endo**	**exo**	**endo**	**exo**	**endo**	**exo**	**endo**	**exo**	**endo**	**exo**	**endo**	**exo**	
IIA1.6	+	–	–	–	–	–	+	–	+	–	–	–	Figure 1
IIAdL1	KO	–	–	–	–	–	+	–	+	–	–	–	Figure 1
IIAdL1-PD-L1	KO	+	–	–	–	–	+	–	+	–	–	–	Figure 1
IIAdL1-PD-L1-CD86KO	KO	+	–	–	–	–	KO	–	+	–	–	–	Figures 1, 2
IIAdL1-PD-L1/CD86^low^	KO	+	–	–	–	–	KO	low	+	–	–	–	Figure 1
IIAdL1-PD-L1/CD86^int^	KO	+	–	–	–	–	KO	int	+	–	–	–	Figure 1
IIAdL1-PD-L1/CD86^high^	KO	+	–	–	–	–	KO	high	+	–	–	–	Figure 1
IIAdL1-PD-L1/FcgRIIB-CD86KO	KO	+	–	–	–	–	KO	–	+	–	–	+	Figure 3
IIAdL1-PD-L1/FcgRIIB/CD86^low^	KO	+	–	–	–	–	KO	low	+	–	–	+	Figure 3
IIAdL1-PD-L1/FcgRIIB/CD86^high^	KO	+	–	–	–	–	KO	high	+	–	–	+	Figure 3
IIAdL1-ICOSLKO	KO	–	–	–	–	–	+	–	KO	–	–	–	Figure 5
IIAdL1-ICOSL	KO	–	–	–	–	–	+	–	KO	+	–	–	Figure 5
IIAdL1-PD-L1-ICOSLKO	KO	+	–	–	–	–	+	–	KO	–	–	–	Figure 6
IIAdL1-PD-L1-ICOSL	KO	+	–	–	–	–	+	–	KO	+	–	–	Figure 6
IIAdL1-PD-L1/FcgRIIB-ICOSLKO	KO	+	–	–	–	–	+	–	KO	–	–	+	Figures 6, 7
IIAdL1-PD-L1/FcgRIIB/ICOSL	KO	+	–	–	–	–	+	–	KO	+	–	+	Figures 6, 7
CH27	+	–	–	–	+	–	+	–	–	–	+	–	Figure 4
CH27TKO	KO	–	–	–	KO	–	KO	–	–	–	+	–	Figure 4
CH27TKO-PD-L1	KO	+	–	–	KO	–	KO	–	–	–	+	–	Figure 4
CH27TKO-PD-L1/CD86	KO	+	–	–	KO	–	KO	+	–	–	+	–	Figure 4

**Table 3:** T cells used in this study.

	**PD-1**		**CD28**		**ICOS**		**PKCθ**		
	**endo**	**exo**	**endo**	**exo**	**endo**	**exo**	**endo**	**exo**	
DO11.10	+	–	+	–	–	–	–^*^	–	Figures 1, 2, 5
DO11.10-CD28KO	+	–	KO	–	–	–	–	–	Figure 2
DO11.10-ICOS	+	–	+	–	–	+	–	–	Figure 5
DO11.10-PKCθ/ICOS	+	–	+	–	–	+	–	+	Figures 5, 6
2B4.11	+	–	+	–	–	–	–^†^	–	Figure 4
primary CD4^+^	–	–	+	–	–	–	+	–	Figures 3, 5, 6
primary CD8^+^	–	–	+	–	–	–	+	–	Figures 3, 5, 6
primary pre-activated CD4^+^	+	–	+	–	+	–	+	–	Figures 3, 4, 6, 7
primary pre-activated CD8^+^	+	–	+	–	+	–	+	–	Figures 3, 4, 6, 7

About

* 1/810 and

† *1/27 of primary CD4^+^ T cells by qPCR*.

### PD-1 Inhibited the Antigen-Dependent Functional Activation of DO11.10 T Cells Less Efficiently in the Presence of CD28 Co-stimulation

DO11.10 T cells endogenously expressed CD28, whereas IIA1.6 cells expressed a low amount of CD86 but did not express CD80 (Figure 1B). We knocked out CD86 gene in IIAdL1-PD-L1 cells to obtain IIAdL1-PD-L1-CD86KO cells (Figure 1E). DO11.10 T cells produced a substantial amount of IL-2 upon stimulation with pOVA_323−339_-pulsed IIAdL1-PD-L1-CD86KO cells in the absence of PD-1 engagement (i.e., in the presence of anti-PD-L1 Ab), indicating that DO11.10 T cells can be activated in the absence of CD28 co-stimulation (Figure 1F). Then, we overexpressed CD86 into IIAdL1-PD-L1-CD86KO cells using weak, intermediate, and strong expression systems to obtain IIAdL1-PD-L1-CD86^low^, CD86^int^, and CD86^high^ cells, respectively (Figure 1E). When we stimulated DO11.10 T cells with these cells expressing different amounts of CD86 in the absence of PD-1 engagement, a good correlation between the amount of secreted IL-2 and the CD86 expression level was observed (*r* = 0.985, Figures 1F,G), indicating that we could regulate the strength of CD28 signal by changing the expression level of CD86 on APCs.

Then we examined the effect of CD28 co-stimulation on PD-1 function. Strong PD-1-mediated suppression of IL-2 production was observed in the absence of CD28 engagement, indicating that PD-1 does not exclusively target CD28 signal but can directly inhibit TCR signal with high efficiency (Figure 1H). PD-1 also suppressed the augmented IL-2 production in the presence of CD28 co-stimulation but the inhibitory efficiency was rather attenuated by CD28 co-stimulation (97 and 80% inhibition with no and strong CD28 engagement, respectively) (Figures 1H,I). By comparing the magnitude of activation and the efficiency of PD-1-dependent inhibition (Figure 1J), we found that PD-1 inhibited T cell activation more efficiently in the absence of CD28 co-stimulation when stimulatory conditions with a similar magnitude of strength (i.e., amount of secreted IL-2) were compared. Thus, the weaker PD-1 effect in the presence of CD28 engagement is not simply due to the vigorous T cell activation with strong CD28 co-stimulation.

**Figure 2:**
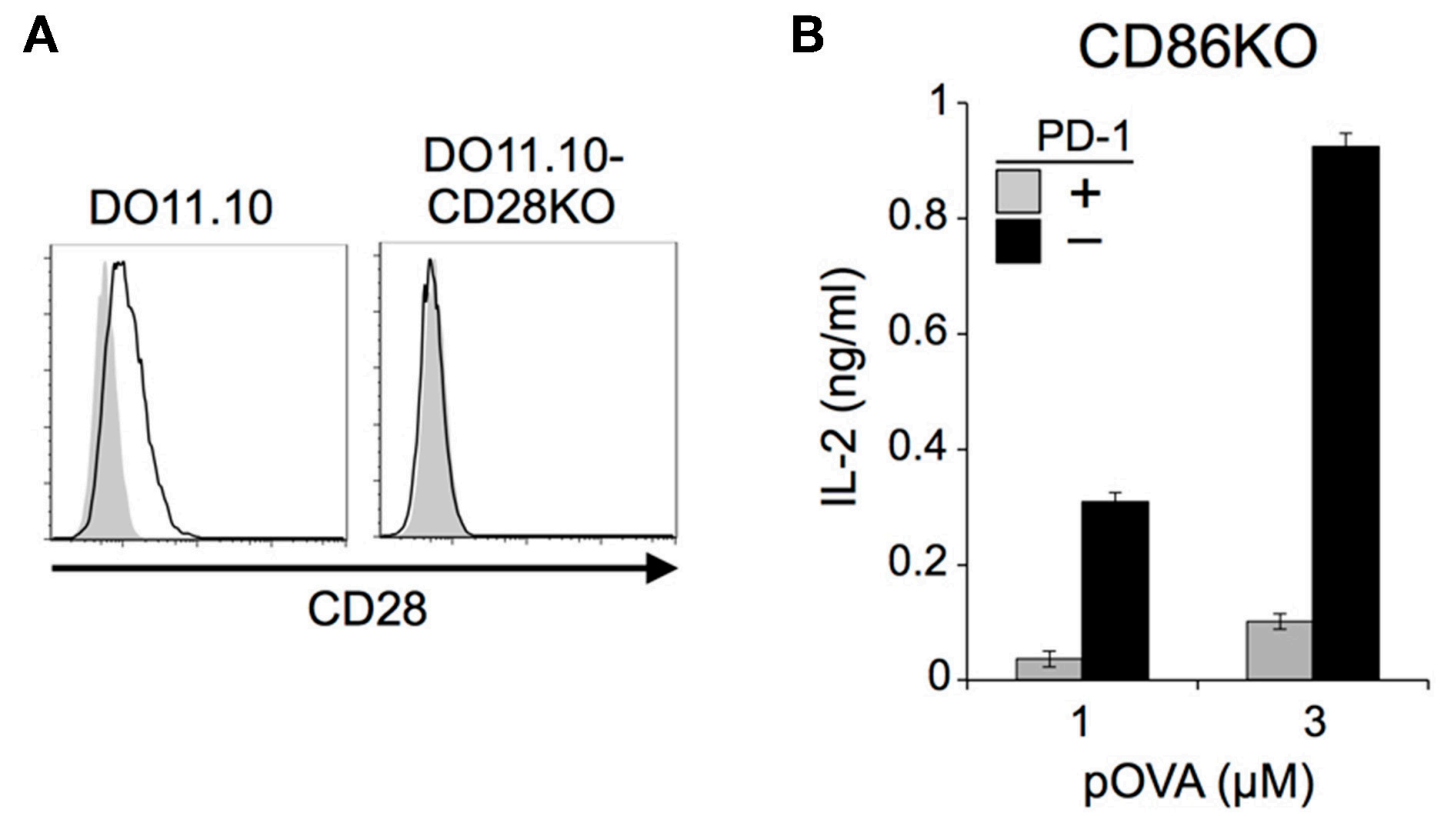
PD-1 inhibited the antigen-dependent functional activation of DO11.10 T cells in the absence of CD28 co-stimulation. **(A)** Expression levels of CD28 on DO11.10 and DO11.10-CD28KO T cells. **(B)** PD-1-dependent inhibition of the antigen-dependent functional activation of DO11.10 T cells without CD28 co-stimulation. IL-2 secretion from DO11.10-CD28KO T cells upon stimulation with pOVA_323−339_-pulsed IIAdL1-CD86KO cells in the presence (gray) or absence (black) of PD-1 engagement. Data are the mean ± SEM of technical triplicates in one experiment. Data are representative of three independent experiments. Cells used in this figure are listed in Tables 2, 3.

We also tested DO11.10-CD28KO T cells to exclude the possible effect of TCR-uncoupled engagement of CD28 in the context of T-T interaction. PD-1 efficiently inhibited IL-2 production from DO11.10-CD28KO T cells (Figure 2; Tables 2, 3), further confirming that PD-1 can inhibit the antigen-dependent functional activation of DO11.10 T cells in the absence of CD28 co-stimulation.

### PD-1 Inhibited the TCR-Dependent Functional Activation of Primary T Cells Less Efficiently in the Presence of CD28 Co-stimulation

**Figure 3:**
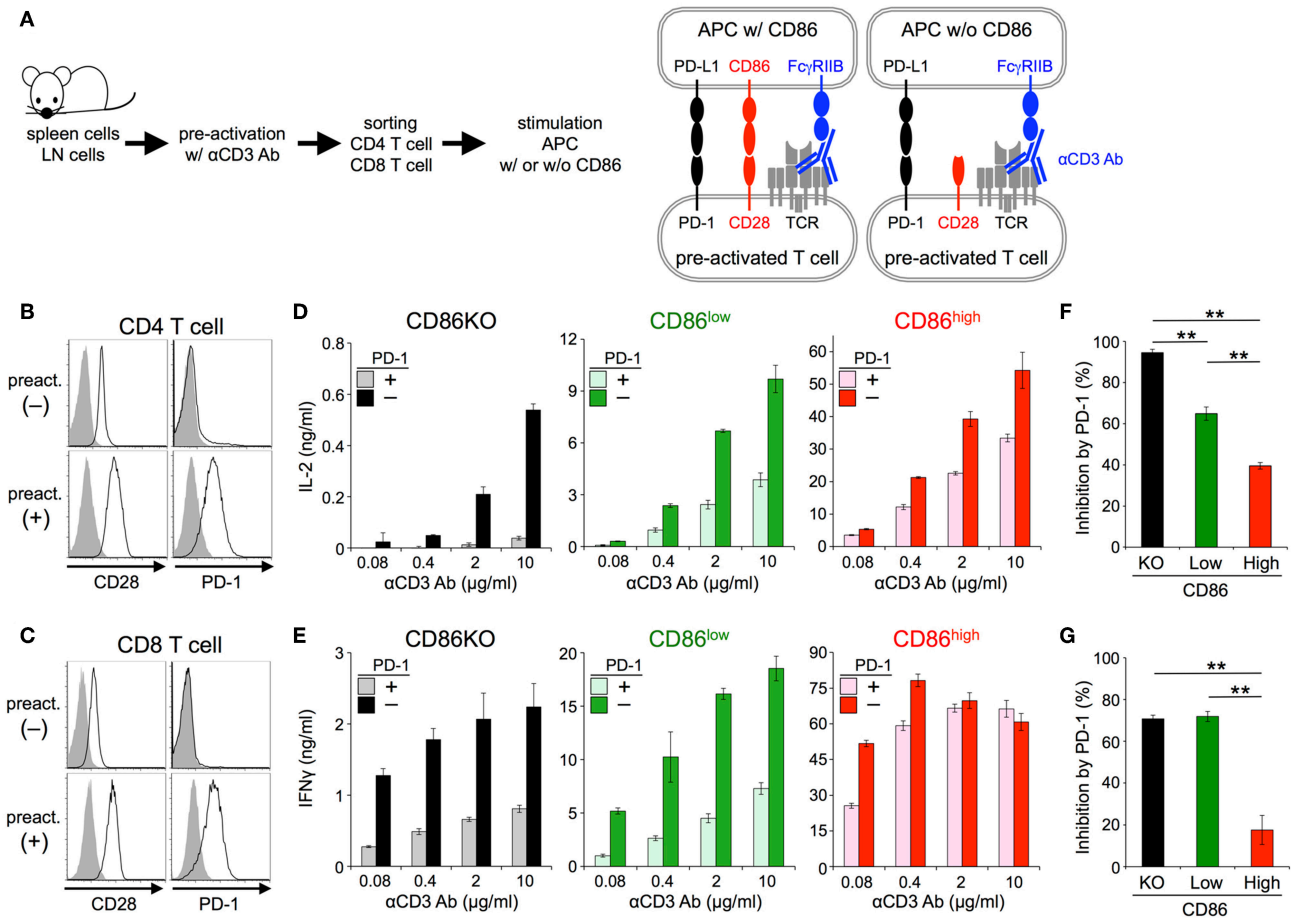
PD-1 inhibited the TCR-dependent functional activation of primary T cells less efficiently in the presence of CD28 co-stimulation. **(A)** Schematic representations of the TCR-dependent activation of primary T cells. Pre-activated CD4^+^ and CD8^+^ T cells were stimulated with anti-CD3 Ab presented on APCs with or without CD86 expression. **(B,C)** Expression levels of CD28 and PD-1 on CD4^+^ **(B)** and CD8^+^ **(C)** T cells with (lower) or without (upper) pre-activation. **(D–G)** Robust PD-1-mediated inhibition of cytokine production from primary T cells in the absence CD28 co-stimulation and the partial attenuation of PD-1-mediated inhibitory effect by CD28 co-stimulation. IL-2 and IFNγ secretions from pre-activated primary CD4^+^ **(D)** and CD8^+^ **(E)** T cells, respectively upon TCR stimulation with no (left, black and gray), weak (middle, green and olive), and strong (right, red and pink) CD28 co-stimulation. PD-1-dependent inhibitory effect was evaluated by comparing amounts of cytokines in the presence (gray, olive, and pink) or absence (black, green, and red) of PD-1 engagement. The average percent PD-1-dependent inhibition of cytokine production upon stimulation with indicated APCs pulsed with 0.08, 0.4, 2, and 10 μg/ml of anti-CD3 Ab is shown for pre-activated primary CD4^+^ **(F)** and CD8^+^ **(G)** T cells. Data are the mean ± SEM of technical triplicates in one experiment. Data are representative of more than two independent experiments. **p < 0.01 by one-way ANOVA with Tukey HSD test. Cells used in this figure are listed in Tables 2, 3.

Then we tested the inhibitory effect of PD-1 in the absence or presence of CD28 co-stimulation using primary T cells (Figure 3A; Tables 2, 3). Because PD-1 is not expressed on naive T cells, we induced PD-1 expression by pre-activation with anti-CD3 Ab (Figures 3B,C). To stimulate pre-activated primary T cells with intact APCs and engage stimulatory and inhibitory co-receptors by native ligands (i.e., not by using plate-bound Ab or truncated proteins tethered to synthetic lipid bilayer), we presented anti-CD3 Ab on APCs overexpressing FcγRIIB (33). We could observe the production of IL-2 and IFNγ from pre-activated primary CD4^+^ and CD8^+^ T cells, respectively in a manner dependent on the amount of anti-CD3 Ab. PD-1 strongly suppressed the production of cytokines from pre-activated primary CD4^+^ and CD8^+^ T cells in the absence of CD28 co-stimulation (Figures 3D,E). As is the case with DO11.10 T cells, the inhibitory efficiency of PD-1 was significantly attenuated in the presence of strong CD28 co-stimulation (95 vs. 40% and 71 vs. 18% inhibition of IL-2 and IFNγ production from CD4^+^ and CD8^+^ T cells, respectively) (Figures 3F,G).

**Figure 4:**
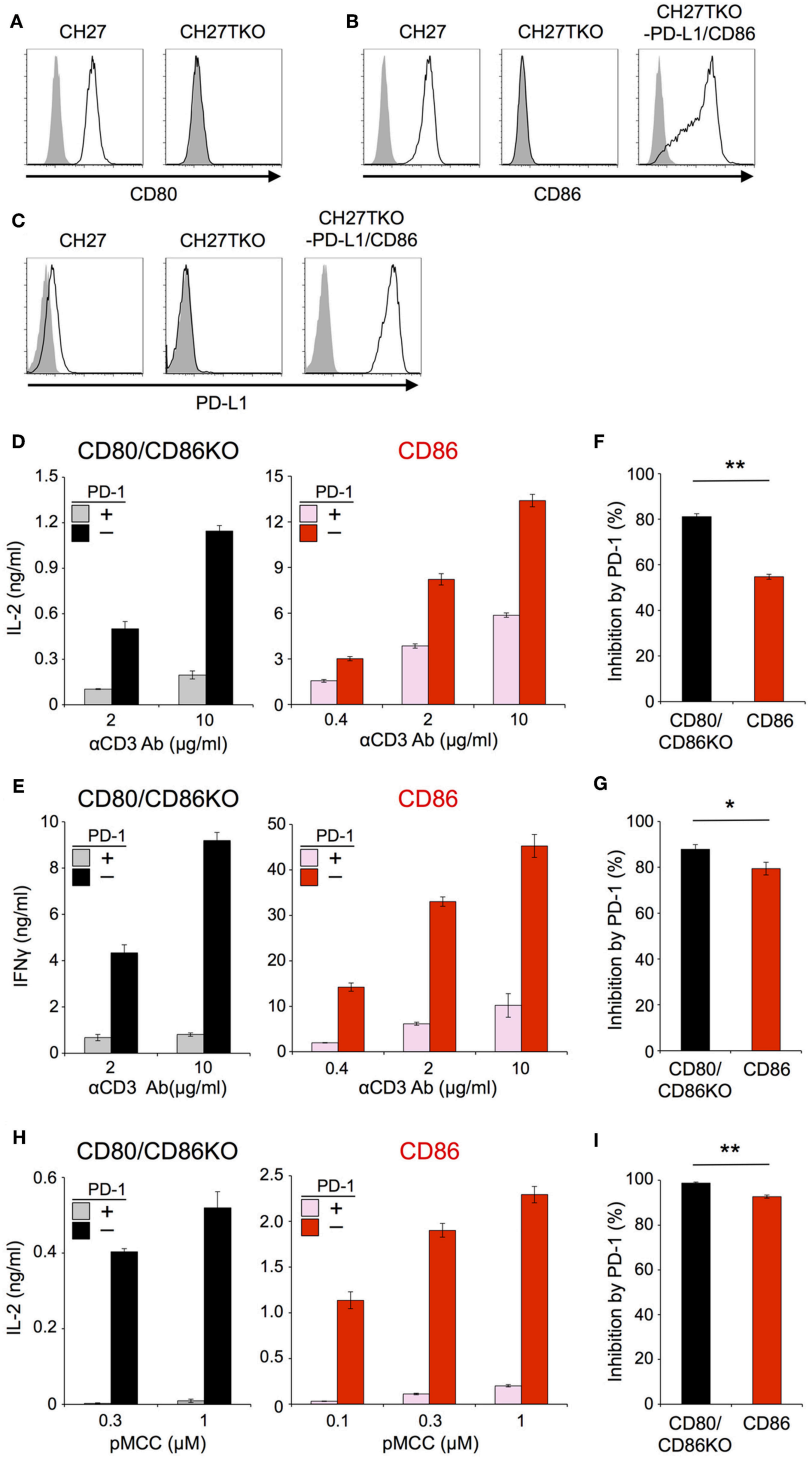
Stimulation of primary T cells using CH27 B lymphoma cells as APCs. **(A–C)** Expression of CD80 **(A)**, CD86 **(B)**, and PD-L1 **(C)** on CH27 cells. CH27 cells endogenously express CD80, CD86, and PD-L1. Genes for CD80, CD86, and PD-L1 were knocked-out in CH27TKO cells. **(D–G)** Robust PD-1-mediated inhibition of cytokine production from primary T cells in the absence of CD28 co-stimulation and the partial attenuation of PD-1-mediated inhibitory effect by CD28 co-stimulation. IL-2 and IFNγ secretion from pre-activated primary CD4^+^ **(D)** and CD8^+^ **(E)** T cells, respectively upon TCR-stimulation in the absence (left, black and gray) or presence (right, red and pink) of CD28 engagement. PD-1-dependent inhibitory effect was evaluated by comparing amounts of cytokines in the presence (gray and pink) or absence (black and red) of PD-1 engagement. The average percent PD-1-dependent inhibition of cytokine production upon stimulation with indicated APCs pulsed with 2 and 10 μg/ml of anti-CD3 Ab is shown for pre-activated primary CD4^+^ **(F)** and CD8^+^ **(G)** T cells. **(H,I)** PD-1-dependent inhibition of the antigen-dependent functional activation of 2B4.11 T cells without CD28 co-stimulation. CH27TKO cells with (right, red and pink) or without (left, black and gray) CD86 were pulsed with indicated amounts of pMCC and used to stimulate 2B4.11 T cells. IL-2 secretion in the presence (gray and pink) or absence (black and red) PD-1 engagement is shown **(H)**. The percent PD-1-dependent inhibition denotes the average inhibitory effects with 0.3 and 1 μM of pMCC **(I)**. Data are the mean ± SEM of technical triplicates in one experiment. Data are representative of two independent experiments. *p < 0.05 and **p < 0.01 by two-tailed unpaired Student's *t*-test. Cells used in this figure are listed in Tables 2, 3.

In order to exclude the possibility that the CD28-independency of PD-1 function is due to an unknown unique property of IIA1.6 cells, we performed similar experiments using another B lymphoma cells, CH27 cells as APCs. Because CH27 cells expressed CD80, CD86, and PD-L1, we knocked out corresponding genes by using CRISPR/cas9 system to obtain cells lacking CD80, CD86, and PD-L1 (CH27TKO cells). Then we overexpressed PD-L1 and CD86 into CH27TKO cells and used them as APCs to stimulate pre-activated CD4^+^ and CD8^+^ T cells (Figures 4A–C; Tables 2, 3). PD-1 efficiently inhibited the production of IL-2 and IFNγ from pre-activated primary CD4^+^ and CD8^+^ T cells, respectively in the absence of CD28 co-stimulation. As is the case with IIA1.6 cells, PD-1 effects were significantly attenuated in the presence of CD28 co-stimulation when CH27 cells were used as APCs (Figures 4D–G). We could also observe similar trends in PD-1 effects on the activation of 2B4.11 T hybridoma cells upon stimulation with CH27TKO cells pulsed with the cognate antigen (88-103 segment of moth cytochrome c, pMCC_88−103_) in the presence or absence of CD28 co-stimulation (Figures 4H,I). These results further support the idea that PD-1 can directly inhibit TCR signal and that CD28 co-stimulation rather attenuates the inhibitory efficiency of PD-1 on TCR-dependent functional T cell activation.

### PD-1 Efficiently Inhibited the TCR-Dependent Functional Activation of T Cells in the Presence of ICOS Co-stimulation

**Figure 5:**
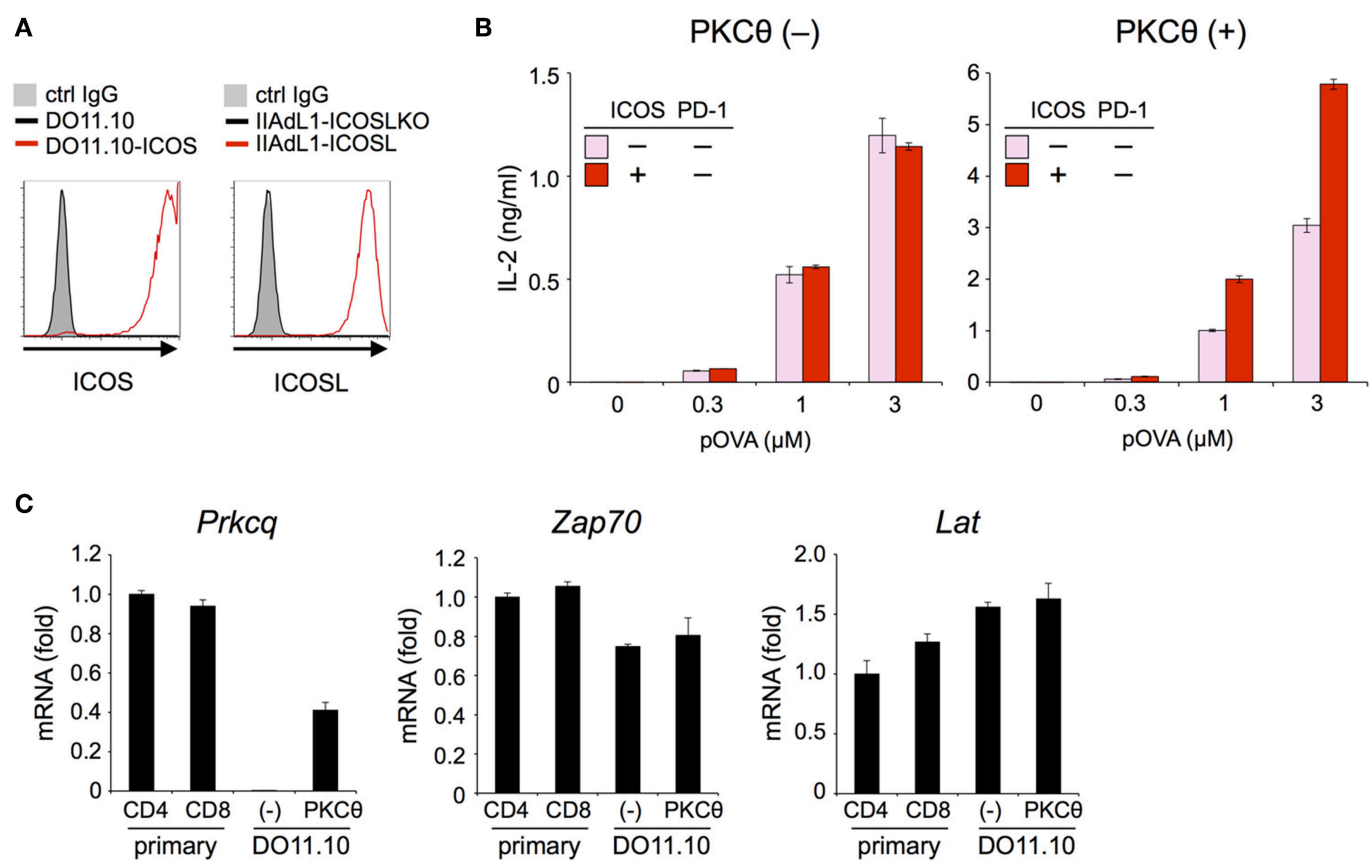
ICOS engagement augmented the antigen-dependent functional activation of DO11.10 T cells in the presence of PKCθ. **(A)** Expression levels of ICOS and ICOSL. **(B)** Increased antigen-dependent secretion of IL-2 from DO11.10-ICOS T cells by ICOS engagement in the presence of PKCθ. ICOS engagement increased the IL-2 secretion from DO11.10-ICOS T cells overexpressing PKCθ (right) but not from DO11.10-ICOS T cells lacking PKCθ expression (left). **(C)** Lack of *Prkcq* (PKCθ) expression in DO11.10 T cells. The expression levels of *Prkcq, Zap70*, and *Lat* in primary CD4^+^ and CD8^+^ T cells and DO11.10 T cells with or without PKCθ overexpression were quantified by qPCR. Fold changes in expression relative to primary CD4^+^ T cells are shown. Data are the mean ± SEM of technical triplicates in one experiment. Data are representative of two independent experiments. Cells used in this figure are listed in Tables 2, 3.

**Figure 6:**
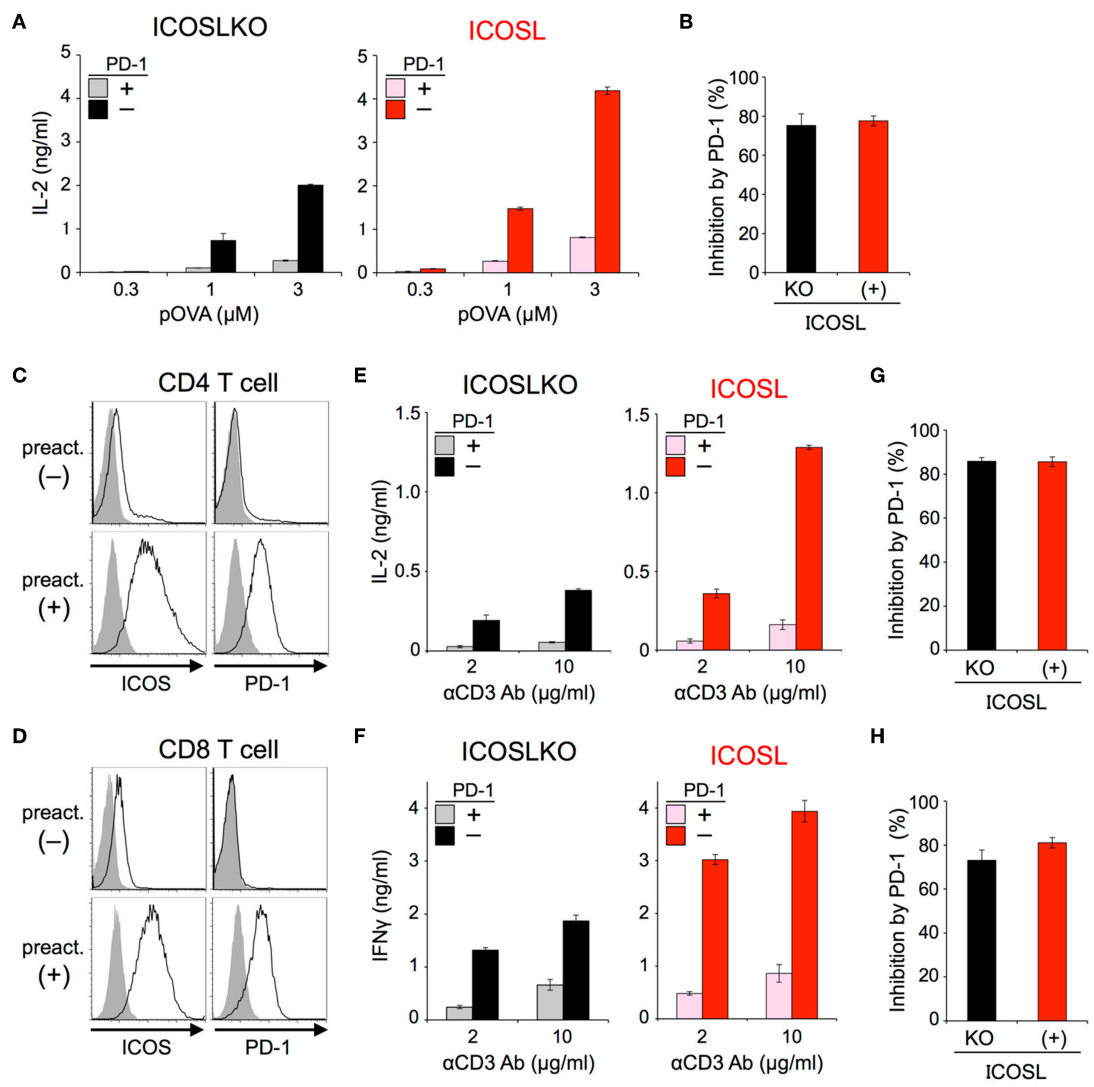
PD-1 efficiently inhibited the functional activation of DO11.10 and primary T cells upon TCR stimulation with ICOS co-stimulation. **(A,B)** PD-1-dependent inhibition of the functional activation of DO11.10 T cells upon antigen stimulation with ICOS co-stimulation. IL-2 secretion from DO11.10 T cells upon stimulation with pOVA_323−339_-pulsed APCs lacking (left, black and gray) or expressing (right, red and pink) ICOSL in the presence (gray and pink) or absence (black and red) of PD-1 engagement **(A)**. The average percent PD-1-dependent inhibition of IL-2 production upon stimulation with indicated APCs pulsed with 0.3, 1, and 3 μM of pOVA_323−339_ **(B)**. **(C,D)** Expression levels of ICOS and PD-1 on CD4^+^ **(C)** and CD8^+^ **(D)** T cells with (lower) or without (upper) pre-activation. **(E–H)** PD-1-dependent inhibition of the functional activation of primary T cells upon TCR stimulation with ICOS co-stimulation. IL-2 and IFNγ secretion from pre-activated primary CD4^+^ **(E)** and CD8^+^ **(F)** T cells, respectively upon TCR stimulation in the absence (left, black and gray) or presence (right, red and pink) of ICOS co-stimulation. PD-1-dependent inhibitory effect was evaluated by comparing amounts of cytokines in the presence (gray and pink) or absence (black and red) of PD-1 engagement. The average percent PD-1-dependent inhibition of cytokine production upon stimulation with indicated APCs pulsed with 2 and 10 μg/ml of anti-CD3 Ab is shown for pre-activated primary CD4^+^ **(G)** and CD8^+^ **(H)** T cells. Data are the mean ± SEM of technical triplicates in one experiment. Data are representative of more than two independent experiments. Cells used in this figure are listed in Tables 2, 3.

The inhibitory capacity of PD-1 against TCR-dependent activation of T cells with ICOS co-stimulation is also controversial (14, 15). Therefore, we examined the inhibitory effect of PD-1 on the functional activation of T cells in the presence of ICOS co-stimulation. Because IIA1.6 cells expressed the ligand of ICOS (ICOSL), we knocked out the gene encoding ICOSL to obtain IIAdL1-ICOSLKO cells and subsequently overexpressed ICOSL (IIAdL1-ICOSL cells). We overexpressed ICOS in DO11.10 T cells because its expression was lacking (Figure 5A; Tables 2, 3). ICOS-engagement augmented IL-2 production from DO11.10 T cells in a PKCθ-dependent manner in agreement with former reports that ICOS co-stimulation substantially augmented IL-2 production (Figures 5B,C) (34–36). Then we examined PD-1 effect and found that PD-1 could strongly inhibit the IL-2 production from DO11.10 T cells upon antigen stimulation with ICOS co-stimulation (Figure 6A; Tables 2, 3). In contrast to CD28, the inhibitory efficiency of PD-1 was not reduced by ICOS co-stimulation (Figure 6B).

Then we tested the inhibitory effect of PD-1 against TCR-dependent activation of primary T cells with ICOS co-stimulation. Because ICOS as well as PD-1 is not expressed on naive T cells, we induced the expression of ICOS and PD-1 by pre-activation with anti-CD3 Ab (Figures 6C,D). When we stimulated pre-activated CD4^+^ and CD8^+^ T cells using APCs with or without ICOSL, ICOS engagement augmented the production of IL-2 and IFNγ from CD4^+^ and CD8^+^ T cells up to 3.4 and 2.3-folds, respectively. PD-1 strongly suppressed the production of cytokines from pre-activated primary CD4^+^ and CD8^+^ T cells in the presence of ICOS co-stimulation (Figures 6E,F). The inhibitory efficiency of PD-1 in the presence or absence of ICOS co-stimulation was also comparable in the activation of primary T cells (Figures 6G,H).

### PD-1 Restricted the Maintenance of Antigen-Induced T_FH_ Cells That Required ICOS Co-stimulation

**Figure 7:**
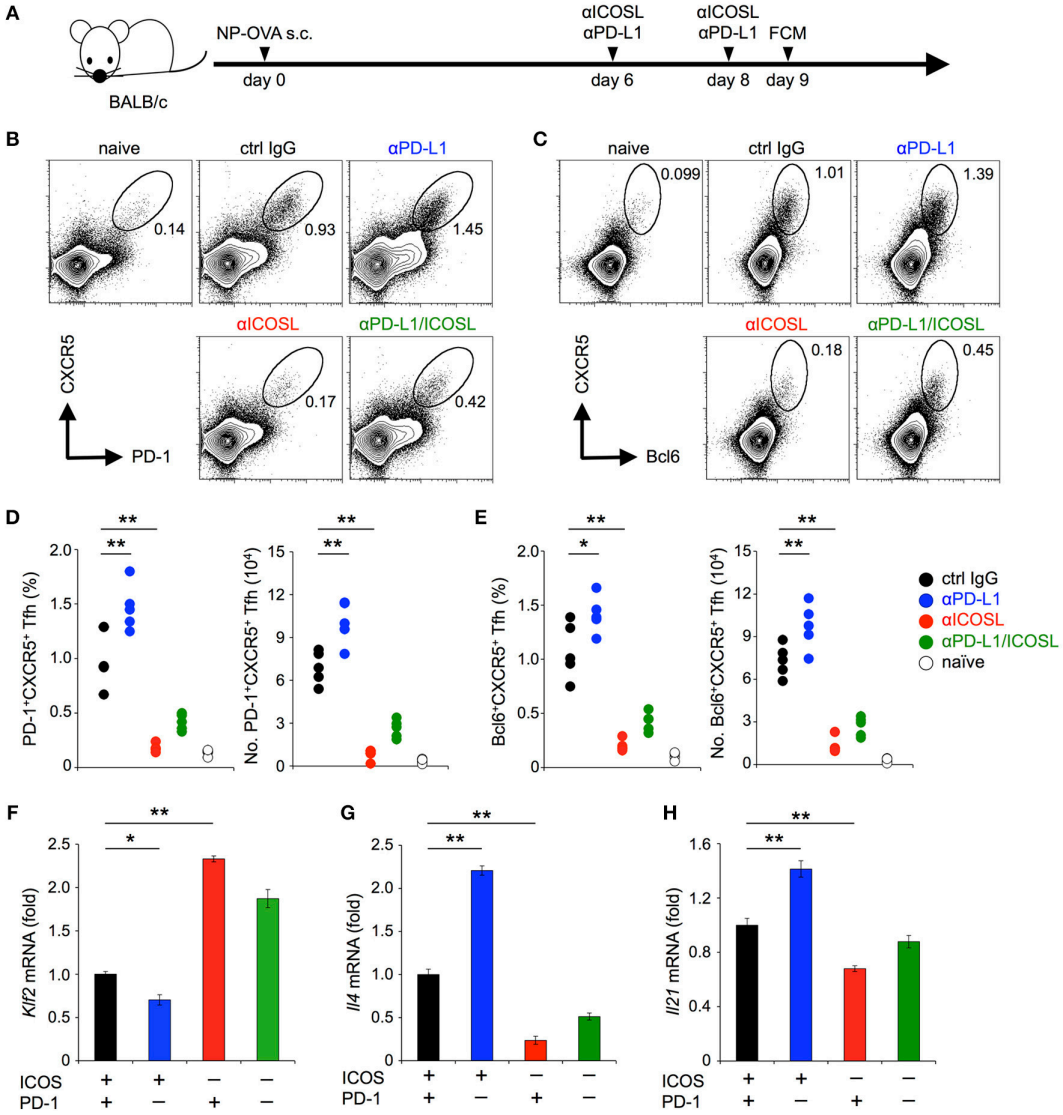
PD-1 restricted the maintenance of antigen-induced T_FH_ cells that required ICOS co-stimulation. **(A)** Schematic representation of the experimental system to analyze PD-1 effects on the maintenance of antigen-induced T_FH_ cells. Anti-ICOSL and anti-PD-L1 Abs were administrated independently or in combination on days 6 and 8 to analyze their effects on the maintenance of antigen-induced T_FH_ cells. CD4^+^ T cells in draining lymph nodes were analyzed on day 9. **(B–E)** Increase of T_FH_ cells by PD-1 blockade during their maintenance phase. Representative flowcytometric profiles **(B,C)** and statistics **(D,E)** of T_FH_ cells identified by the expression of CXCR5 and PD-1 **(B,D)** and CXCR5 and Bcl6 **(C,E)** are shown for mice with the indicated treatment. **(F–H)** PD-1 blockade facilitated the down-regulation of *Klf2* **(F)** and up-regulation of *Il4* **(G)** and *Il21* **(H)** upon TCR stimulation with ICOS co-stimulation. Pre-activated primary CD4^+^ T cells were stimulated with anti-CD3 Ab (2C11, 0.1 μg/ml) in the indicated combination of ICOS and PD-1 engagements for 6 h. T cells were purified and the expression of *Klf2, Il4*, and *Il21* was quantified by qPCR. Data are the mean ± SEM of technical triplicates in one experiment. Data are representative of more than two independent experiments. *p < 0.05 and **p < 0.01 by one-way ANOVA with Tukey HSD test. Cells used in this figure are listed in Tables 2, 3.

The antigen-dependent generation of T_FH_ cells requires CD28 co-stimulation (37–39). On the other hand, their maintenance requires ICOS but not CD28 co-stimulation (40–44). PD-1 is highly expressed on T_FH_ cells and has been shown to regulate the function of T_FH_ cells (37). In order to confirm that PD-1 can inhibit the functional T cell activation upon TCR signal co-stimulated by ICOS *in vivo*, we examined the effect of PD-1 on the maintenance of T_FH_ cells. We immunized mice with NP-OVA, a T-dependent antigen to induce germinal center reaction (Figure 7A). In agreement with the former report (44), the administration of anti-ICOSL blocking Ab during the maintenance phase of T_FH_ cells (6 and 8 days after immunization) markedly decreased the frequency and the number of T_FH_ cells (1.0 vs. 0.18 % and 6.9 vs. 1.2 × 10^4^ cells for control IgG- and anti-ICOSL Ab-treated mice, respectively). In contrast, the administration of anti-PD-L1 blocking Ab during the maintenance phase of T_FH_ cells significantly increased the frequency and the number of T_FH_ cells (1.0 vs. 1.5 % and 6.9 vs. 10.1 × 10^4^ cells for control IgG- and anti-PD-L1 Ab-treated mice, respectively) (Figures 7B–E). These results suggest that the maintenance of T_FH_ cells by TCR signal co-stimulated by ICOS is persistently weakened by PD-1 to restrict the number of T_FH_ cells, which is in agreement with former reports that T_FH_ cells with lower affinity were increased in the absence of PD-1 (45–47).

### PD-1 Partially Attenuated the Down-Regulation *Klf2* Upon TCR Stimulation With ICOS Co-stimulation

The maintenance of T_FH_ cells by ICOS has been reported to depend on the facilitation of TCR-dependent reduction of Kruppel-like factor 2, a zinc-finger transcription factor (KLF2, *Klf2*) by ICOS co-stimulation (44, 48). Therefore, we examined the effect of PD-1 on the expression of *Klf2* by stimulating pre-activated CD4^+^ T cells in conditions involving PD-1 and/or ICOS (Figures 7F–H; Tables 2, 3). In agreement with the former report, we observed the facilitation of TCR-dependent reduction of *Klf2* expression by ICOS engagement (44). In addition, ICOS engagement augmented the TCR-dependent up-regulation of *Il21* and *Il4*, which are required for the function of T_FH_ cells. Remarkably, PD-1 blockade in the presence of ICOS co-stimulation further facilitated the TCR-dependent down-regulation of *Klf2* and up-regulation of *Il21* and *Il4*, in accordance with the increase of T_FH_ cells by PD-1 blockade *in vivo* (Figures 7F–H). Therefore, PD-1 likely restricts the generation of T_FH_ cells by restraining the down-regulation of *Klf2* upon TCR stimulation with ICOS co-stimulation during the germinal center reaction.

## Discussion

In the current study, by taking the advantage of the *in vitro* and *ex vivo* co-culture systems of T cells and APCs and the genome-editing technology, we directly tested the molecular coordination of PD-1 and stimulatory co-receptors in the functional activation of T cells. We clearly demonstrated that PD-1 could inhibit cytokine production from T cells upon activation in the absence as well as in the presence of CD28 co-stimulation. Meanwhile, PD-1 inhibited functional T cell activation with ICOS co-stimulation as efficiently as that with CD28 co-stimulation. In addition, we found that the maintenance of T_FH_ cells that depended on TCR signal co-stimulated by ICOS was persistently restrained by PD-1 *in vivo*. Stimulatory co-receptors depend on TCR signal for their activation and function by reinforcing TCR signal (1, 3). Therefore, the most straightforward interpretation of aforementioned results is that PD-1 primarily inhibits TCR signal. Thus, PD-1 functions as a rheostat of T cell activation rather than an inhibitor of a specific stimulatory co-receptor. Intriguingly, the inhibitory efficiency of PD-1 was substantially lower in the presence of CD28 co-stimulation, suggesting that the CD28 signaling pathway is rather resistant to PD-1 and/or CD28 co-stimulation renders TCR signal resistant to PD-1.

Bennet et al. reported that PD-1 could inhibit T cell activation with ICOS but not CD28 co-stimulation (15). On the other hand, Hui et al. reported that PD-1 dephosphorylated CD28 but not CD3ζ and ICOS using a cell-free assay system. The preferential dephosphorylation of CD28 by PD-1 was also demonstrated in Jurkat cells in the latter study. However, the dephosphorylation of CD28 was observed only transiently and CD28 was phosphorylated to a similar level in the absence or presence of PD-1 by 10 min after TCR stimulation (14). T cell activation is a complex multistep process and the longer and/or serial encounter of antigens over the period of hours and days is required for the functional activation of T cells (19–23). Thus, the transient and the moderate difference in the phosphorylation level of CD28 may not represent the actual effect of PD-1 on the functional activation of T cells. In addition, the target specificity may not be preserved in the cell-free assay system enough. Actually, CD45, a receptor-type protein tyrosine phosphatase that is known to dephosphorylate Y505 of Lck at the early phase of T cell activation (49) dephosphorylated broader targets and showed similar preference to CD28 in the cell-free assay (14). Although CD45 is segregated from the center of the immune synapse during T cell activation (50, 51), the segregation might not occur properly in the cell-free assay.

In the current study, by using the acquisition of cytokine production capacity that directly reflects T cell activation and represents one of the most important functions of T cells as an unambiguous benchmark of functional T cell activation, we clearly demonstrated that PD-1 did not exclusively target CD28 signal and that CD28 co-stimulation was rather prohibitive for PD-1 function.

In line with the preferential dephosphorylation of CD28 by PD-1 in the aforementioned cell free assay (14), Kamphorst et al. demonstrated that the blockade of CD80/CD86 attenuated the therapeutic effects of anti-PD-L1 Ab against CT26 colon tumor cells (52). Although their observation clearly supports the conclusion that CD28-dependent co-stimulation is required for the re-activation of PD-1^+^ T cells by PD-1 blockade, it does not necessarily argue for the inhibition of CD28 signal by PD-1 or deny the inhibition of TCR signal by PD-1 in the eradication of tumor cells (18). It should also be noted that CT26 cells strongly express CD80 (53), and thus, might be affected by CD28-blockade more strongly than tumor cells without CD80 expression. Meanwhile, current findings do not preclude the requirement of CD28 co-stimulation in the re-activation of exhausted PD-1^+^ T cells.

The recent success of cancer immunotherapies targeting PD-1 and the development of autoimmune diseases as their immune related adverse events revealed that PD-1 plays critical roles in the regulation of T cells specific to self antigens as well as tumor-associated antigens in humans. Extensive trials of combinatorial therapies targeting multiple inhibitory and stimulatory co-receptors are ongoing to increase the efficacy of cancer immunotherapies while minimizing immune related adverse events. Precise and deeper understandings of the inhibitory mechanisms of PD-1 will support the rational development of immuno-modulatory therapies for a diverse array of diseases.

## Data Availability

All data sets generated for this study are included in the manuscript.

## Ethics Statement

This study was carried out in accordance with the recommendations of the Regulation for Animal Experiment Management at Tokushima University by the Animal Experimentation Committee of Tokushima University. The protocol was approved by the Animal Experimentation Committee of Tokushima University.

## Author Contributions

RM, DS, IO, and TO designed the experiments. RM, DS, KS, TM, MW, and IO established experimental systems, performed the staining and functional experiments using cultured and primary cells. RM and DS generated gene-targeted cells. RM, IO, and TO wrote the manuscript with all authors contributing to writing. TO oversaw the entire project.

### Conflict of Interest Statement

The authors declare that the research was conducted in the absence of any commercial or financial relationships that could be construed as a potential conflict of interest.

## Abbreviations

APC: antigen-presenting cell
ICOS: inducible T cell co-stimulator
ICOSL: ligand of ICOS
KLF2: Kruppel-like factor 2
PD-1: Programmed cell death 1
TCR: T cell receptor
T_FH_: follicular helper T.
